# Short Association Fibres of the Insula-Temporoparietal Junction in Early Psychosis: A Diffusion Tensor Imaging Study

**DOI:** 10.1371/journal.pone.0112842

**Published:** 2014-11-18

**Authors:** Sean N. Hatton, Jim Lagopoulos, Daniel F. Hermens, Ian B. Hickie, Elizabeth Scott, Maxwell R. Bennett

**Affiliations:** Clinical Research Unit, Brain & Mind Research Institute, University of Sydney, NSW, Australia; Chiba University Center for Forensic Mental Health, Japan

## Abstract

Evidence shows that there are reductions in gray matter volume (GMV) and changes in long association white matter fibres within the left insula-temporoparietal junction (TPJ) during the early stages of psychotic disorders but less is known about short association fibres (sAFs). In this study we sought to characterise the changes in sAFs and associated volumetric changes of the left insula-TPJ during the early stages of psychosis. Magnetic resonance imaging was obtained from a sample of young people with psychosis (n = 42) and healthy controls (n = 45), and cortical parcellations of the left insula-TPJ were used as seeding masks to reconstruct 13 sAFs. Compared to healthy counterparts, the psychosis group showed significant reductions in fractional anisotropy (FA) in the sAFs connecting the superior (STG) and middle temporal gyri (MTG) and as well as reduced GMV within the inferior temporal gyrus and increased white matter volume (WMV) within Heschl's gyrus (HG). Furthermore, adolescent-onset psychosis subjects (onset 18 year or earlier) showed FA reductions in the STG-HG sAF when compared to adult-onset subjects, but this was not associated with changes in GMV nor WMV of the STG or HG. These findings suggest that during the early stages of psychosis, changes in sAFs and associated cortical GMV and WMV appear to occur independently, however age of onset of a psychotic syndrome/disorder influences the pattern of neuroanatomical abnormalities.

## Introduction

Short association fibres (sAFs) comprise either local association fibres (U-fibres of Mynert) [1] that arch through the cortical sulci to connect adjacent gyri or neighbouring association fibres that connect nearby cortical gyri [2], as opposed to long association fibres that connect distant cortical regions [3]. Our previous investigations have shown that in the early stages of psychosis there is reduced cortical thickness within the left temporo-parietal junction (TPJ) [4] and altered white matter (WM) cohesion within the left inferior and superior longitudinal fasciculi associated with deficits in attention and verbal fluency [5]. Furthermore, we have reported that young people at the early stages of psychiatric disorders (psychosis, bipolar, depression and anxiety disorders) have reduced left anterior insula gray matter volume (GMV) that was correlated with worse attention-set shifting [6]. Collectively, this evidence suggest that in the early stages of psychosis, deficits in attention and verbal fluency are underpinned by left anterior insula GMV reductions and reduced cortical thickness of the left TPJ without involvement of the left uncinate that connects the two regions. These findings are supported by a variety of neuropathological and neuroimaging evidence of left insula-TPJ abnormalities in psychoses. Neuropathological investigations show that reduced surface areas of the left planum temporale and Heschl's gyrus was found in psychosis subjects relative to controls [7] and that the normal hemispheric asymmetry of the supramarginal gyrus (SMG), with larger neurons and marginally lower neuron density in the left hemisphere, is less evident in psychosis subjects [8]. Diffusion-weighted imaging demonstrated that WM tracts tended to be larger only in the left hemisphere [9], and it has been proposed that atypical patterns of brain lateralization in this region give rise to the abnormalities between thoughts and expression seen in psychosis [10]–[12]. Furthermore, compared to controls, the functional connectivity of the default mode network between the posterior corpus callosum and bilateral TPJ was significantly decreased in early schizophrenia subjects (mean age 25.9 years), and this connectivity reduced with increasing duration of illness [13]. To further understand the relationship between neurostructural abnormalities in the left hemisphere that underpin cognitive deficits in young people at the early stages of psychosis, we sought to characterise the sAFs that connects the left insula-TPJ.

Superficial cerebral WM is comprised of striatal, thalamic, pontine and commissural fibres as well as long and short association fibres [3]. A recent report [14] analysed the effects of age (18–74 years), gender and asymmetry of superficial WM in a healthy population, showing that with age, all lobes showed a progressive reduction in the ease of diffusivity along WM pathways as indicated by a reduction in fractional anisotropy (FA). Conversely, age-related increases in axial diffusivity (AD), a marker of axonal coherence, and radial diffusivity (RD), a marker of myelin integrity, was observed across the entire brain with the exception of the inferior temporal pole. The report also highlights that neurotypical superficial WM has an asymmetrical distribution, with FA being higher in the left frontal, temporal and parietal regions and right medial occipital region. Mirroring this asymmetry, AD and RD was increased in the right temporal, parietal, and lateral occipital lobes as well as the middle and superior frontal gyrus. The authors suggest that these distributions of superficial WM are indicative of higher left hemisphere axonal density and myelination in keeping with a similar report of greater fibre coherence and myelination within the left perisylvian regions of healthy young people [15]. An earlier report by the same group [16] found that superficial WM FA was significantly reduced in the left temporal lobes in subjects with adult-onset schizophrenia (mean age of onset 22.2 years) compared to healthy controls, and that there were no associations between this neuroanatomical change and symptom severity. A subsequent investigation of superficial WM in older schizophrenia subjects (mean age 36 years) [17] identified five clusters within the left hemisphere that exhibited reduced FA compared to matched controls, and three-quarters of the voxels within these clusters were associated with sAFs rather than deep long association fibres. Interestingly, the largest cluster encompassing the left occipital region, precuneus and posterior cingulate cortex is attributed to the default mode network [18], and a smaller cluster encompassing the frontal operculum and insula is attributed with the attentional switch between the default mode network and attentional control network [19]. Collectively, these studies indicate that superficial WM FA within the left insula-TPJ is altered in older psychosis subjects and may be associated with attentional deficits, though the evidence of such neuroanatomical changes at earlier stages of psychosis remains unanswered.

The principle aim of this study was to delineate sAFs in young healthy individuals and compare these with sAFs in young people at the early stages of psychosis. Based on previous research into superficial WM changes in older psychosis subjects [16], [17], we hypothesized that sAFs integrity will be impaired in the temporal regions of younger psychosis subjects relative to the controls. To further understand this effect, we also chose to compare sAFs integrity between controls, adolescent-onset psychosis (onset 18 years or younger) and adult-onset psychosis subjects (onset 19 years or older). Secondarily, we sought to understand if changes in sAFs affect the GMV or WMV of connected gyri. Considering the distinct asymmetry of temporal superficial WM between hemispheres [14], [15], this study only concentrated on left hemispheric structures.

## Material and Methods

### Subjects and clinical assessment

Building on our recent investigation into long association fibres in early psychosis [5], the present study used forty-two outpatients aged 15 to 34 years and forty-five healthy control subjects recruited from specialist youth mental health clinics in Sydney Australia [20]. Exclusion criteria for all subjects included medical instability (as determined by a psychiatrist), history of neurological disease (e.g. tumour, head trauma, epilepsy), medical illness known to impact cognitive and brain function (e.g. cancer), intellectual and/or developmental disability, insufficient English for neuropsychological assessment and current substance dependence. Participants with hazardous levels of alcohol consumption (as determined by the Alcohol Use Disorders Identification Test) or a history of excessive substance abuse (i.e. substance-induced psychotic disorder) were excluded from this present investigation. All subjects were asked to abstain from drug or alcohol use for 48 hours prior to testing. The University of Sydney ethics committee approved the study and subjects gave written informed consent prior to participation in the study. Parents or guardians provided written informed consent for subjects under the age of 16 years. Psychosis subjects were assessed by a psychiatrist to be of adequate competency to provide informed consent during recruitment. All psychosis subjects were outpatients that were not floridly psychotic during any stage of this investigation and were stabilised on their medication.

A psychiatrist assessed all patients to determine the nature and history of any mental health problems. By consensus of the senior investigators (IBH and ES), subjects were categorized according to DSM-IV-TR criteria [21]. We collectively analysed schizophrenia (n = 12) schizophreniform (n = 15), schizoaffective (n = 7) and psychosis not otherwise specified (NOS, n = 7) together. Among patients, nine subjects (21%) were not taking any psychotropic medications at the time of testing, whereas 12 (29%) were taking a second-generation antidepressant, 31 (74%) an atypical antipsychotic medication and four (10%) a mood stabiliser. Of the 11 (26%) subjects taking multiple medications, seven (64%) were taking antidepressant and antipsychotic medication, one (9%) was taking antipsychotic and mood stabilising medication, and three (27%) were taking a combination of antidepressant, antipsychotic and mood stabilising medications. At the time of assessment no control subjects were taking any psychotropic medications.

A psychiatrist or trained research psychologist conducted the clinical assessment (in a semi-structured interview format) to determine the nature and history of any mental health problems using the Brain and Mind Research Institute Structured Interview for Neurobiological Studies [22]. The assessment included the Hamilton Depression Rating Scale (HDRS) [23] to quantify mood symptoms and the Brief Psychiatric Rating Scale (BPRS) [24] to quantify current general psychiatric symptom severity as well as positive and negative symptoms. Premorbid intelligence (“predicted IQ”) was estimated from the Wechsler Test of Adult Reading [25]. A self-report assessment was also completed by all subjects that included the Kessler-10 (K-10) [26] to assess psychological distress as well as the Depression Anxiety and Stress Scales (DASS) [27] to assess depression, anxiety, and tension/stress.

### Magnetic resonance imaging acquisition and analysis

Subjects underwent MRI scanning using a 3-Tesla GE MR750 Discovery scanner (GE Medical Systems, Milwaukee, WI) at the Brain and Mind Research Institute, Camperdown, NSW Australia. To enable neuroanatomical analysis at high resolution (0.9 mm isotropic resolution), we acquired a customized MP-RAGE 3D T1-weighted sequence: repetition time (TR) = 7264 msec; echo time (TE)  = 2784 msec; flip angle  = 15; coronal orientation; field of view (FOV)  = 230 mm; matrix of 256×256; total slices  = 196. Next, whole brain diffusion-weighted images were acquired using an echo planar imaging sequence: TR  = 7000 ms; TE  = 68 ms; slice thickness  = 2.0 mm; FOV = 230×230 mm; acquisition matrix  = 256×256; axial orientation; 69 gradient directions. Eight images without gradient loading (b = 0 s/mm^2^) were acquired prior to the acquisition of 69 images (each containing 55 slices) with uniform gradient loading (b = 1159 s/mm^2^).

### White matter tractography

Using FSL version 5.0 [28]–[30], diffusion-weighted volumes were eddy current corrected and brain tissue was extracted. A diffusion tensor model was fitted at each voxel using DTIFIT and each resulting tensor map was inspected for the appropriate reconstruction of the major pathways.

**Figure 1:**
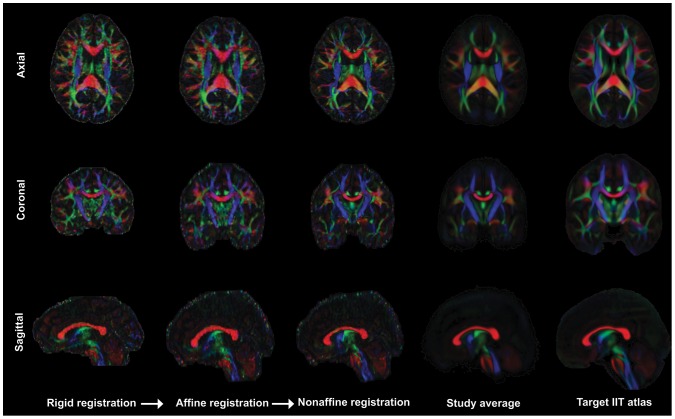
Example tensor-based registration of an individual subject to the IIT atlas. Each individual's DTI data was aligned to the Illinois Institute of Technology (IIT) Human Brain Atlas using the tensor-based registration tool DTI-TK [32], [33]. Tensor images were aligned to the IIT/ICBM standard space in a three stages – four iterations of rigid registration, four iterations of affine registration, then five iterations of diffeomorphic, non-affine registration. Throughout this process each individual mask was checked for artifacts, full coverage of the brain and correct orientation.

To provide accurate mapping of the sAFs across subjects, each individual's DTI data was aligned to the Illinois Institute of Technology (IIT) Human Brain Atlas (version 3) [31] using the tensor-based registration tool DTI-TK [32], [33]. Full tensor images were iteratively aligned to the IIT/ICBM standard space as follows (Figure 1): initially a rigid registration using Euclidean Distance Squared metrics with four iterations and ftol  = 0.01, then an affine registration using Euclidean Distance Squared metrics with four iterations and ftol  = 0.01, and finally a diffeomorphic, non-affine registration with five iterations and ftol  = 0.002.

### Reconstruction of short association fibres

To create seeding targets for reconstructing sAF, cortical gyri demarcated by the Desikan–Killiany atlas [34] were extracted using the FreeSurfer software package version 5.1 (http://surfer.nmr.mgh.harvard.edu/) as previously described [35]. These seeding regions were linearly registered to the IIT/ICBM standard space using FLIRT [36], [37] with 12 DOF, mutual information cost function and spline interpolation. This process was then followed by a non-linear registration using FNIRT using the T1_2_MNI152_2mm configurations provided within FSL 5.0, but using the IIT Human Brain Atlas as the reference image and mask.

Using DTI-TK, the FA scalar information from the IIT Atlas was extracted and a binary WM map was built with a lower threshold of 0.1 to capture the detail of these finer tracts. Nine cortical parcellations from the Desikan–Killiany atlas were used as inclusion targets to delineate the sAF within the left insula-TPJ, namely the superior temporal gyrus (STG), transverse temporal gyrus (i.e. Heschl's gyrus, HG), supramarginal gyrus (SMG), inferior parietal lobe (IPL), fusiform (FUS), inferior temporal gyrus (ITG), middle temporal gyrus (MTG), the banks of the superior temporal sulcus (BANKS) and the insula (INSULA). Tracking was performed using two gyri as inclusion regions of interest (ROIs) with the remaining seven gyri used as exclusion ROIs. To further remove extraneous superficial fibres (i.e. striatal, thalamic, pontine, commissural and long association fibres) from sAFs, exclusion ROIs of the lateral occipital and midline were also applied and stray fibres were manually removed using ITK-SNAP. Pairing of some ROIs resulted in less than 50 streamlines per connection and were subsequently excluded from the study.

Whereas long association fibre templates are widely available, the present study required the creation of sAFs templates (Figures S1–13). This was accomplished using the continuous medial representation framework, a 3D geometrical model that defines the skeleton and boundaries of brain structures [38]. From each of the binarized sAFs were generated a speed, initialization and levelset function to construct an initial boundary of the sAF. From this boundary was created the skeleton of the sAF using the application defaults with the exception of the pruning factor: all sAFs used a pruning factor of p = 1.4 with the exception of the ITG-FUS sAF which used p = 2.5. Within Matlab R2012a for Linux a medial representation of this skeleton was generated using a Laplace Basis mapping technique with the following iterative stages: alignment with blur  = 0.24; affine registration with blur  = 0.24, 400 iterations; first deformation with blur  = 0.18, 2000 iterations; second deformation with blur  = 0.12, 4000 iterations; third and final deformation with blur  = 0.12, 6000 iterations. Medial templates that gave a Dice similarity coefficient of 88% or greater were considered an adequate representation of the sAF [39].

### Extraction of individual volumetrics

To determine the GMV and WMV of each ROI, FreeSurfer software package version 5.1 [35] was use to parcellate the left insula-TPJ of each individual, and full details of the FreeSurfer process for this cohort have been previously reported [4].

### Statistical analysis

Statistical analyses were performed using the Statistical Package for the Social Sciences (SPSS 22.0 for Windows). Between groups demographic analyses were conducted using chi-square tests for categorical data. One-way analysis of variance (ANOVA) with posthoc Bonferroni pairwise comparisons was used to assess differences in age, predicted IQ, HDRS, K10 and DASS. The age of onset of psychosis, duration of illness and BPRS scores was assessed using independent-samples *t*-tests. Volumetric differences were evaluated using an MANCOVA analysing intracranial-corrected ROI volumes between groups, controlling for age and gender. Significance was set at *p*<0.05 (two-tailed).

Within the DTI-TK program we used the tract-specific analysis generalised linear model with 10,000 permutations to determine group differences in FA, AD and RD measurements. Significance was set at *p*<0.05 which converted to a *t*-statistic threshold of 1.66.

## Results

### Demographic and clinical scores

There were no significant group differences in handedness or predicted IQ between controls, adolescent-onset psychosis and adult-onset psychosis subjects (Table 1). Collectively, the psychosis subjects contained significantly more males (psychosis 71.4% vs. controls 44.4%) and were younger than their healthy counterparts (22.7±3.9 years; 24.8±3.4 years, respectively). There were significantly more males in the adult-onset psychosis group than control group (*p* = .018) and the adolescent-onset group was significantly younger age (19.7 years mean, 3.6 SD) than the adolescent-onset group (24.1 years mean, 3.2 SD, *p*<.001) or control group (24.8 years mean, 3.4 SD, *p*<.001). The psychosis subjects had significantly worse HDRS, K10 and DASS clinical scores than controls.

**Table 1:** Demographics and clinical symptom scores (mean ± SD).

Demographic/clinical variable	Control (*n* = 45)	Adolescent-onset (*n* = 13)	Adult-onset (*n* = 29)	Significance (df) [*p*]	Comment
Male/female	20/25	9/4	21/8	χ^2^(2) = 6.61 [.039]	Controls ≠ Adult-onset
Handedness (r/l/a)	36/9/0	10/2/1	0/1/0	χ^2^(4) = 8.17 [.086]	
Age	24.8±3.4	19.7±3.6	24.1±3.2	*F*(2,84) = 11.47 [<.001]	Adolescent-onset <Controls, Adult-onset
Predicted IQ	103.9±7.8	101.0±9.7	104.4±6.6	*F*(2,47) = 0.45 [.642]	
HDRS	2.9±2.5	15.8±5.6	14.4±7.8	*F*(2,53) = 39.31 [<.001]	Controls <Adolescent-onset, Adult-onset
K10	15.7±4.5	27.5±5.9	23.1±9.4	*F*(2,75) = 18.92 [<.001]	Controls <Adolescent-onset, Adult-onset
DASS Depression scale	4.9±7.1	19.1±11.5	15.2±11.5	*F*(2,78) = 15.64 [<.001]	Controls <Adolescent-onset, Adult-onset
DASS Anxiety scale	3.9±5.7	17.8±8.1	12.9±11.2	*F*(2,77) = 16.99 [<.001]	Controls <Adolescent-onset, Adult-onset
DASS Stress scale	7.8±7.7	19.1±8.4	15.3±9.4	*F*(2,78) = 11.37 [<.001]	Controls <Adolescent-onset, Adult-onset
Age of onset of psychosis (years)		15.9±1.6	22.3±3.2	*t*(40) = −6.9 [<.001]	Adolescent-onset <Adult-onset
Duration of illness (years)		3.9±3.4	1.7±1.6	*t*(14.5) = 2.2 [.044]	Adult-onset <Adolescent-onset
BPRS Total		47.8±11.2	43.3±12.5	*t*(32) = 1.03 [.312]	
BPRS Positive Symptoms		15.8±6.1	12.6±4.9	*t*(32) = 1.66 [.107]	
BPRS Negative Symptoms		7.1±2.8	9.2±4.2	*t*(32) = −1.53 [.137]	

Gender and handedness were evaluated using a Pearson Chi-square test. One-way analysis of variance (ANOVA) with posthoc Bonferroni pairwise comparisons was used to assess differences in age, predicted IQ, HDRS, K10 and DASS. The age of onset of psychosis, duration of illness and BPRS scores was assessed using independent-samples *t*-tests. Significance levels were set at *p*<.05 (2-tailed). BPRS, Brief Psychiatric Ratings Scale; DASS, Depression Anxiety and Stress Scales; HDRS, Hamilton Depression Rating Scale; K10, Kessler-10; SD, standard deviation.

The mean age of onset of psychosis was 17.6 years (SD 4.2) with a mean duration of illness of 5.2 years (SD 4.0). The adolescent-onset and adult-onset psychosis subjects did not differ in HDRS, K10, DASS or BPRS scores but did differ in age of onset (adolescent-onset group were younger than the adult-onset psychosis group, *p*<.001) and duration of illness (adolescent-onset group had a mean duration of 3.9 years compared to 1.7 years for the adult-onset psychosis group, *p* = .044).

### Group differences in short association fibres

A total of 13 sAFs were constructed as shown in Figure 2. Compared to healthy counterparts, psychosis subjects show a cluster within the STG-MTG (173 mm^2^) that exhibited reduced FA (*t* = 1.95, *p* = 0.036; Figure 3). Within the STG-MTG, there were 18 small clusters (i.e. *t*>1.66) of reduced AD in psychosis subjects compared to controls, but none reached levels of significance (Figure S1), and no significant RD clusters were identified. All other sAFs exhibited several clusters of interest (i.e. *t*>1.66) but none reached levels of significance (full details provided in Figures S2-13).

**Figure 2:**
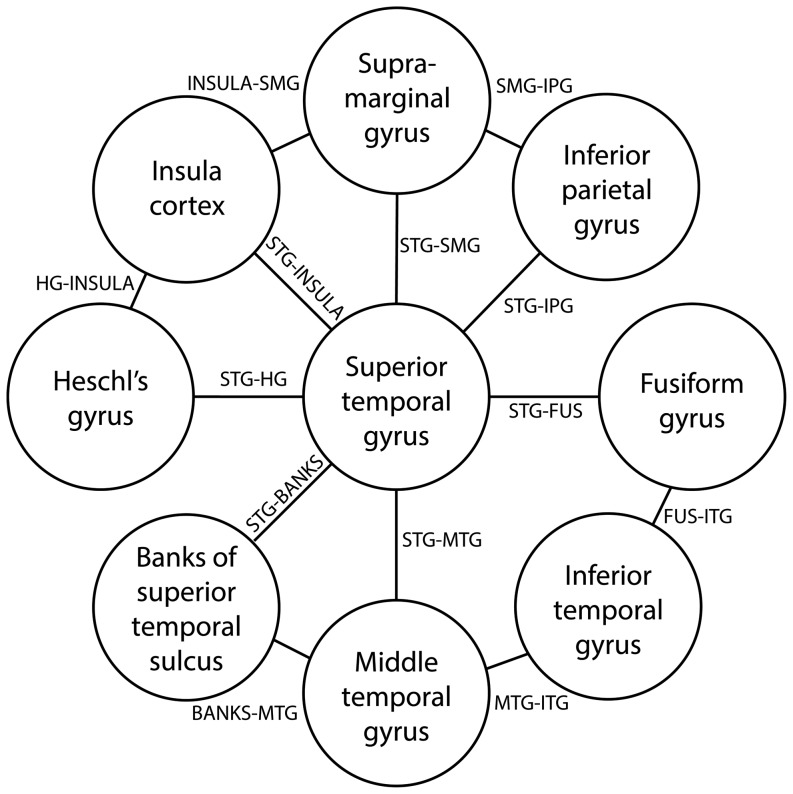
Atlas of the insula-temporoparietal junction.

**Figure 3:**
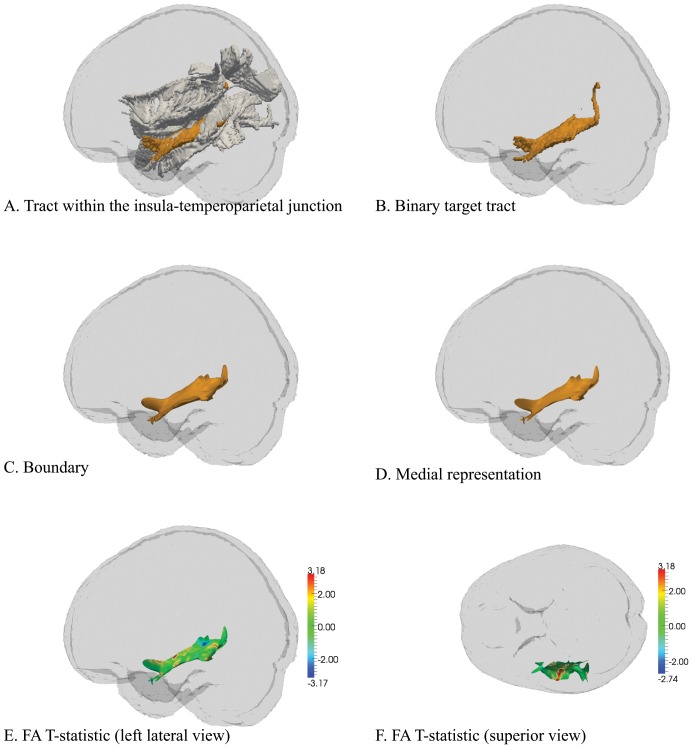
Reduced FA in the short association fibres connecting the left superior and middle temporal gyrus (STG-MTG). A) Lateral perspective of the left insula-TPJ highlighting the STG-MTG short association fibre. B) The STG-MTG binary image isolated. C) The calculated average boundary of the STG-MTG. D) The medial representation of the STG-MTG. E, F) T-statistic maps indicating reduced FA within the STG-MTG in psychosis subjects compared to controls (*p* = 0.036).

Compared to controls, the adolescent-onset psychosis subjects showed reduced FA within the STG-HG (area 107 mm^2^; *t* = 2.01, *p* = 0.047) as well as showing a trend of reduced FA within the STG-MTG (area 154 mm^2^; *t* = 2.00, *p* = 0.051). Within the STG-HG and STG-MTG there were several small clusters (i.e. *t*>1.66) of reduced AD in adolescent-onset psychosis subjects compared to controls, but none of these reached levels of significance, with no RD clusters found. There were no statistically significant differences in the 13 sAF reconstructions between adult-onset psychosis subjects and healthy counterparts.

### Group differences in parcellated volumes

Comparing GMVs between cohorts, the overall MANCOVA did not show a main effect (F_1,86_ = 0.6, *p* = 0.747) but subsequent pairwise comparisons of simple effects showed that left ITG mean GMV was reduced in the psychosis group relative to the control group (12040 mm^3^ vs. 12578 mm^3^, respectively; *p* = 0.044; Table 2). Contrasting adolescent-onset psychosis, adult-onset psychosis and control groups found no significant differences in the GMVs of ROIs examined (Table 3).

**Table 2:** Volumetric differences between control and psychosis groups.

	Mean GMV (mm^3^ ± SD)			Mean WMV (mm^3^ ± SD)		
Region	Control	Psychosis	*p*	Control	Psychosis	*p*
Heschl's gyrus	1295±191	1389±347	ns	913±166	982±173	0.004
Insula cortex	7048±606	7083±721	ns	9091±794	8958±844	ns
Supramarginal gyrus	11975±1374	11974±1462	ns	9056±1012	8914±1078	ns
Inferior parietal cortex	14402±1698	14157±1607	ns	10422±1173	10371±1299	ns
Fusiform gyrus	11320±1213	11233±1100	ns	7145±714	7019±597	ns
Inferior temporal gyrus	12578±1258	12040±1568	0.044	6615±646	6514±818	ns
Middle temporal gyrus	11436±1277	11457±1268	ns	5669±746	5630±661	ns
Superior temporal gyrus	12558±1129	12735±1117	ns	8215±835	7991±809	ns
Banks of superior temporal gyrus	2728±447	2704±543	ns	3107±570	3031±551	ns

Pairwise volumetric comparisons of ROIs in the left insula-TPJ between control (*n* = 45) and psychosis (*n* = 42) groups corrected for intracranial volume, gender and age. GMV, gray matter volume; ns, not significant; SD, standard deviation; WMV, white matter volume.

**Table 3:** Volumetric differences between control, adolescent-onset psychosis, adult-onset psychosis groups.

	Mean GMV (mm^3^ ± SD)				Mean WMV (mm^3^ ± SD)			
Region	Control	Adolescent-onset	Adult-onset	*p*	Control	Adolescent-onset	Adult-onset	*p*
Heschl's gyrus	1295±191	1336±220	1413±392	ns	913±166	939±183	1002±168	0.018 (C<A)
Insula cortex	7048±606	7266±648	7001±748	ns	9091±794	8921±582	8975±947	ns
Supramarginal gyrus	11975±1374	12323±1311	11817±1520	ns	9056±1012	8895±1000	8923±1128	ns
Inferior parietal cortex	14402±1698	14566±1465	13974±1658	ns	10422±1173	10485±1192	10320±1362	ns
Fusiform gyrus	11320±1213	11569±979	11083±1134	ns	7145±714	7060±683	7000±567	ns
Inferior temporal gyrus	12578±1258	12273±2032	11935±1339	ns	6615±646	6639±791	6458±837	ns
Middle temporal gyrus	11436±1277	11944±1421	11239±1154	ns	5669±746	5243±495	5803±659	0.050 (E<A)
Superior temporal gyrus	12558±1129	12888±880	12667±1217	ns	8215±835	7978±907	7997±778	ns
Banks of superior temporal gyrus	2728±447	2706±536	2704±555	ns	3107±570	3010±438	3041±601	ns

Volumetric comparisons of ROIs in the left insula-TPJ between control (C; *n* = 45), adolescent-onset psychosis (E; *n* = 13) and adult-onset psychosis (A; *n* = 29) groups corrected for intracranial volume, gender and age. GMV, gray matter volume; ns, not significant; SD, standard deviation; WMV, white matter volume.

Comparing WMVs between groups, the overall MANCOVA did not show a main effect (F_1,86_ = 1.3, *p* = 0.245), but subsequent pairwise comparisons of simple effects showed that the mean left HG WMW was increased in the psychosis group relative to the control group (psychosis mean WMV 982 mm^3^, controls 913 mm^3^, *p* = 0.004; Table 2). Contrasting by onset (Table 3), the adult-onset psychosis group had larger HG WMV compared to controls (controls mean WMV 913 mm^3^; adult-onset psychosis 1002 mm^3^; *p* = 0.018) and the adolescent-onset psychosis group showed significantly reduced MTG WMV compared with the adult-onset psychosis group (adolescent-onset mean WMV 5243 mm^3^; adult-onset psychosis 5803 mm^3^; *p* = 0.050).

## Discussion

This present study shows that of the 13 sAFs that connect regions of the left insula-TPJ, people in the early stages of psychosis have significant FA reductions within the sAFs connecting the STG and MTG relative to controls. MRI meta-analyses of the structural and functional roles of the STG and MTG [40], [41] have implicated these ROIs in auditory processing and the production of auditory hallucinations, respectively [42]. Compared to controls, the psychosis group also showed reduced GMV in the ITG (Table 2) as previously reported in first episode psychosis [43]. We also report that the psychosis group showed increased WMV within HG relative to controls (Table 2), and this trend was predominately influenced by the adult-onset psychosis subjects (Table 3). Earlier investigations suggest that increased HG GMV and WMV are associated with increased frequency of auditory verbal hallucinations in acute chronic psychosis [44]. Collectively, our findings show that at the early stages of psychosis there are subtle WM and GM changes within the left insula-TPJ that affect the STG and MTG associated with auditory processing. Considering that these changes are influenced by the age of onset, pharmacological intervention strategies may need to be tailored to suit the trajectory of illness as well as the neurodevelopmental stage of the individual.

An important finding in this work is the relationship between volumetric changes in structures of the insula-TPJ and variations in diffusion properties in the sAFs connecting these structures. There is a significant increase in HG WMV in the adult-onset psychosis group compared to controls, but no difference seen in the STG-HG sAF connecting the regions. Conversely, there was reduced FA within the STG-HG sAF in the adolescent-onset psychosis group compared to controls without a significant difference in GMV or WMV of the STG or HG. Similarly, compared to controls, the adolescent-onset group showed reduced FA in the STF-MTG sAF without significant differences in the GMV/WMV of the STG or MTG. Furthermore, the late-onset group showed increased MTG WMV relative to the adolescent-onset group, but this was not associated with FA changes in the STG-MTG sAF. However, the finding of a comparative FA reduction needs to be interpreted with caution: one explanation could be that this result reflects a restriction in diffusivity due to pathology (e.g. myelin loss, axon loss), WM disorganisation (e.g. axonal disorganisation) or WM reorganisation (e.g. crossing-fibres, reduced axonal diameter and packing) [45]. What we can surmise is that there are significant reductions in diffusivity in the sAF connecting different regions of the insula-TPJ without concomitant changes in GMV, WMV or vice-versa. Such results raise the question - can sAFs be altered without an effect on associated GMV or WMVs and vice-versa? This could be better understood by following these subjects longitudinally, as well as better characterising how WM tracts interface with cortical regions [46]. Collectively, this present work suggests that during the early stages of psychosis, changes in sAFs and GM/WMVs in the left insula-TPJ may occur independently and differentially depending on the age of onset.

Previous investigations have identified abnormalities in superficial WM within the left temporoparietal and frontal regions [16], [17], and this present work highlights that the link between the STG and MTG is a significant contributor to these abnormalities during the early stages of psychosis. Since sAFs create extensive connections to integrate functionally connected cortical regions, age-related patterns of superficial WM changes are suggested to contribute to fine-tuning selective cognitive and sensory and motor functions during normal brain maturation [47], [48]. Accordingly, degradation of sAFs from adolescence could play a role in developing the neurocognitive deficits present in the prodromal stages prior to frank psychosis. Of note, FA reductions within the superficial WM of the left frontal operculum and insula region was associated with impairments in processing speed in older schizophrenia subjects that approached significance [17]. Future investigations should explore how disruptions in the normal maturation of sAFs could be associated with the common neurocognitive impairments seen in emerging psychiatric disorders.

### Limitations

There are several limitations to the present study that warrant discussion. It is important to note that the continuous medial representation framework that was used to model the sAFs optimally represents ribbon-like structures such as u-fibres. However, the FUS-ITG sAF had a more complex structure that could not be optimally fitted by the model (Dice similarity coefficient 0.75) and thus these findings need to be interpreted with this constraint in mind. Next, to create sAFs that only included streamlines connecting the seed regions without extraneous inclusions, we set strict exclusion seeds that may have excluded genuine streamlines – it is our belief that this would affect only a small number of streamlines per sAF and is preferential to including misleading reconstructions. Additionally, we chose a shape-based technique to find a balance between deterministic tractography that does not accommodate crossing fibres, and probabilistic tractography that will overestimate tracts – future investigations should model at least two directions (preferably three directions) within each voxel to reconstruct estimated pathways in regions of crossing fibres. As a cross-sectional study with 42 psychosis subjects, the study is underpowered to examine the influence of medication. Finally, variations in AD and RD may be a consequence of partial volume effects or underlying tissue structure [49] which may be independent of psychiatric pathology *per se*, and follow-up replications of these techniques in different population samples would help clarify the nature of these changes in illness progression.

## Conclusions

Our findings indicate that in psychosis there is a significant GMV reduction within the left ITG that is accompanied by reduced FA in the sAFs connecting the MTG and STG. Furthermore, in adolescent-onset psychosis there is reduced FA in the sAFs connecting HG and STG independent of volumetric changes in these two structures, and future investigations should elucidate the interface between sAFs and connecting gyri and how these changes relate to disease progression over time.

## Supporting Information

Figure S1 **Short association fibres connecting the superior and middle temporal gyri.**
(TIFF)

Figure S2 **Short association fibres connecting the fusiform to the inferior temporal gyrus.**
(TIFF)

Figure S3 **Short association fibres connecting the insula cortex to Heschl's gyrus.**
(TIFF)

Figure S4 **Short association fibres connecting the banks of the superior temporl sulcus to the middle temporal gyrus.**
(TIFF)

Figure S5 **Short association fibres connecting the insula cortex to the supramarginal gyrus.**
(TIFF)

Figure S6 **Short association fibres connecting the insula cortex to the supramarginal gyrus.**
(TIFF)

Figure S7 **Short association fibres connecting the supramarginal gyrus to the inferior parietal region.**
(TIFF)

Figure S8 **Short association fibres connecting the banks of the superior temporal sulcus to the superior temporal gyrus.**
(TIFF)

Figure S9 **Short association fibres connecting the fusiform to the superior temporal gyrus.**
(TIFF)

Figure S10 **Short association fibres connecting the Heschls' gyrus to the superior temporal gyrus.**
(TIFF)

Figure S11 **Short association fibres connecting the insula cortex to the superior temporal gyrus.**
(TIFF)

Figure S12 **Short association fibres connecting the inferior parietal cortex to the superior temporal gyrus.**
(TIFF)

Figure S13 **Short association fibres connecting the supramarginal gyrus to the superior temporal gyrus.**
(TIFF)
