# Supplementary figures and images for: Short Association Fibres of the Insula-Temporoparietal Junction in Early Psychosis: A Diffusion Tensor Imaging Study
Source: PLoS One. 2014 Nov 18;9(11):e112842. doi: 10.1371/journal.pone.0112842 (PMC4236116; doi:10.1371/journal.pone.0112842)

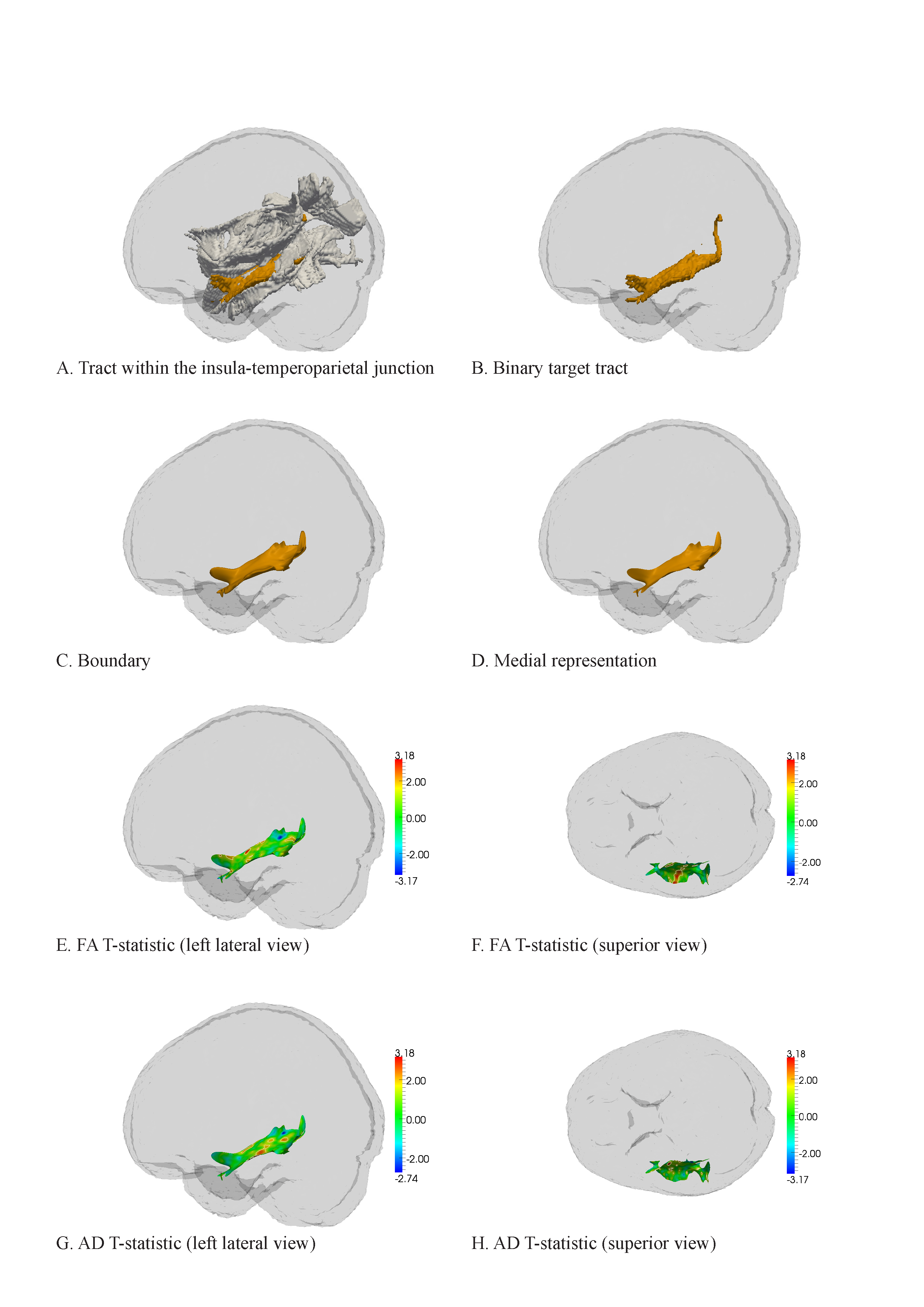

Supplement: Figure S1 — Short association fibres connecting the superior and middle temporal gyri. (TIFF) [file pone.0112842.s001.tiff]

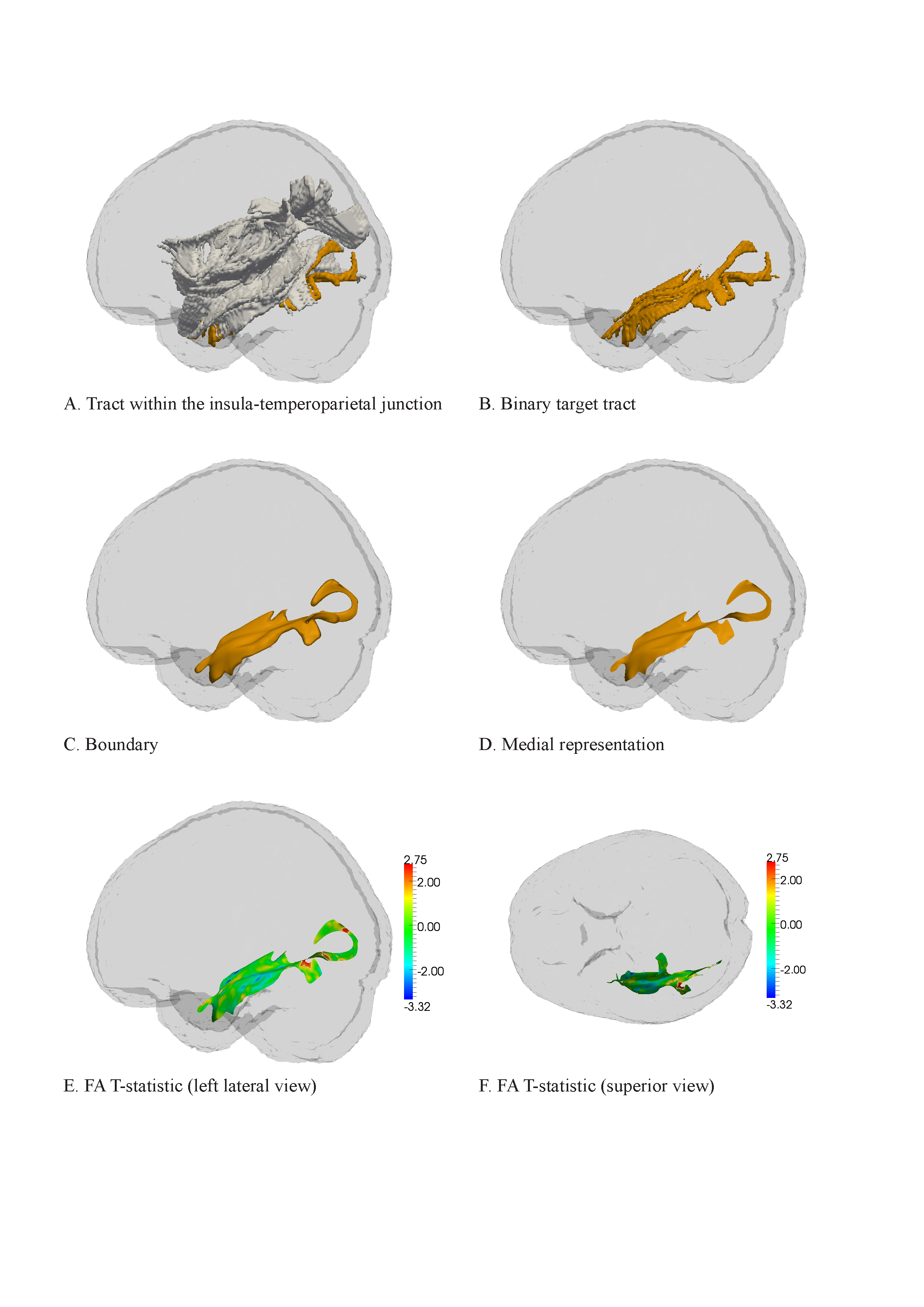

Supplement: Figure S2 — Short association fibres connecting the fusiform to the inferior temporal gyrus. (TIFF) [file pone.0112842.s002.tiff]

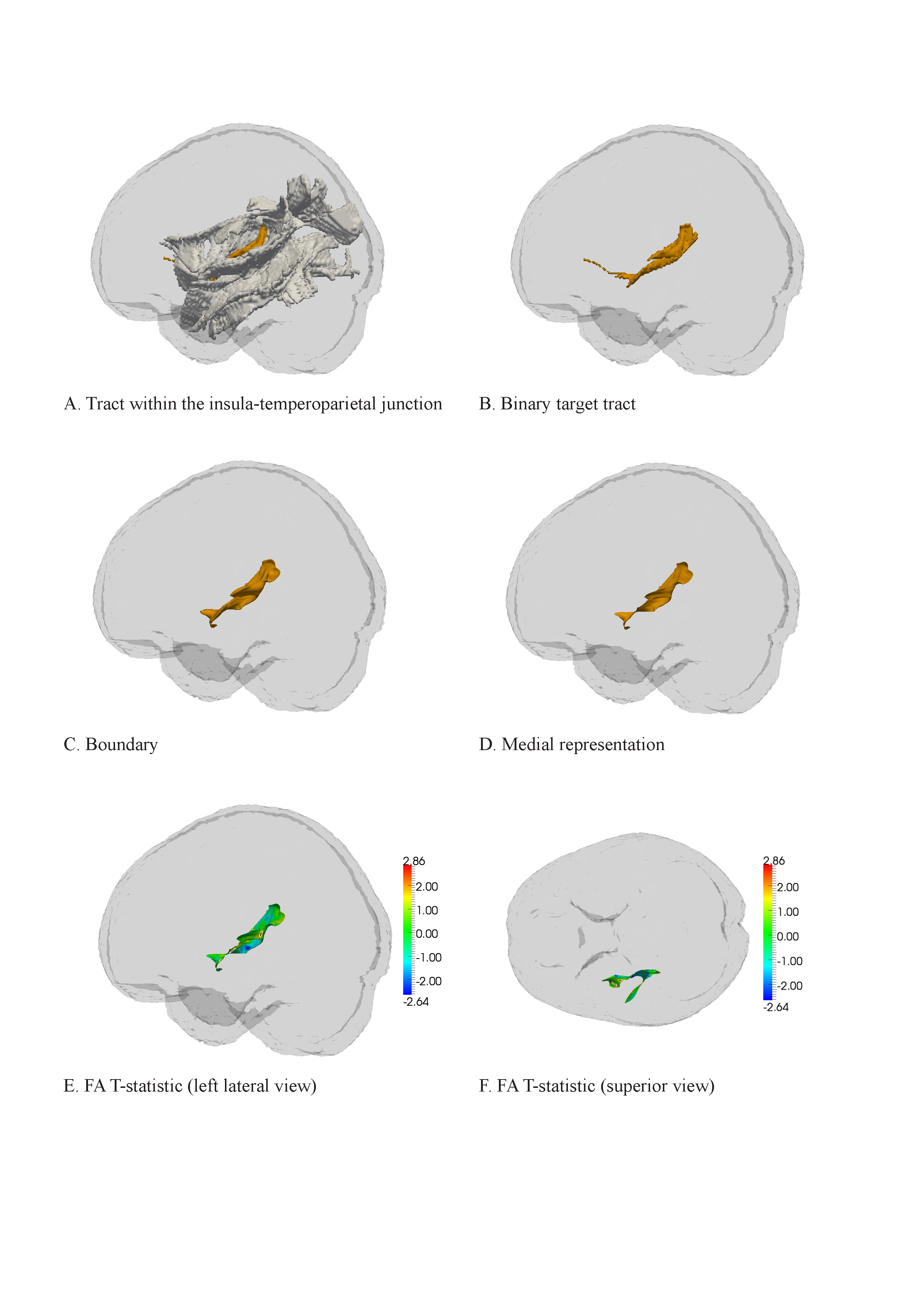

Supplement: Figure S3 — Short association fibres connecting the insula cortex to Heschl's gyrus. (TIFF) [file pone.0112842.s003.tiff]

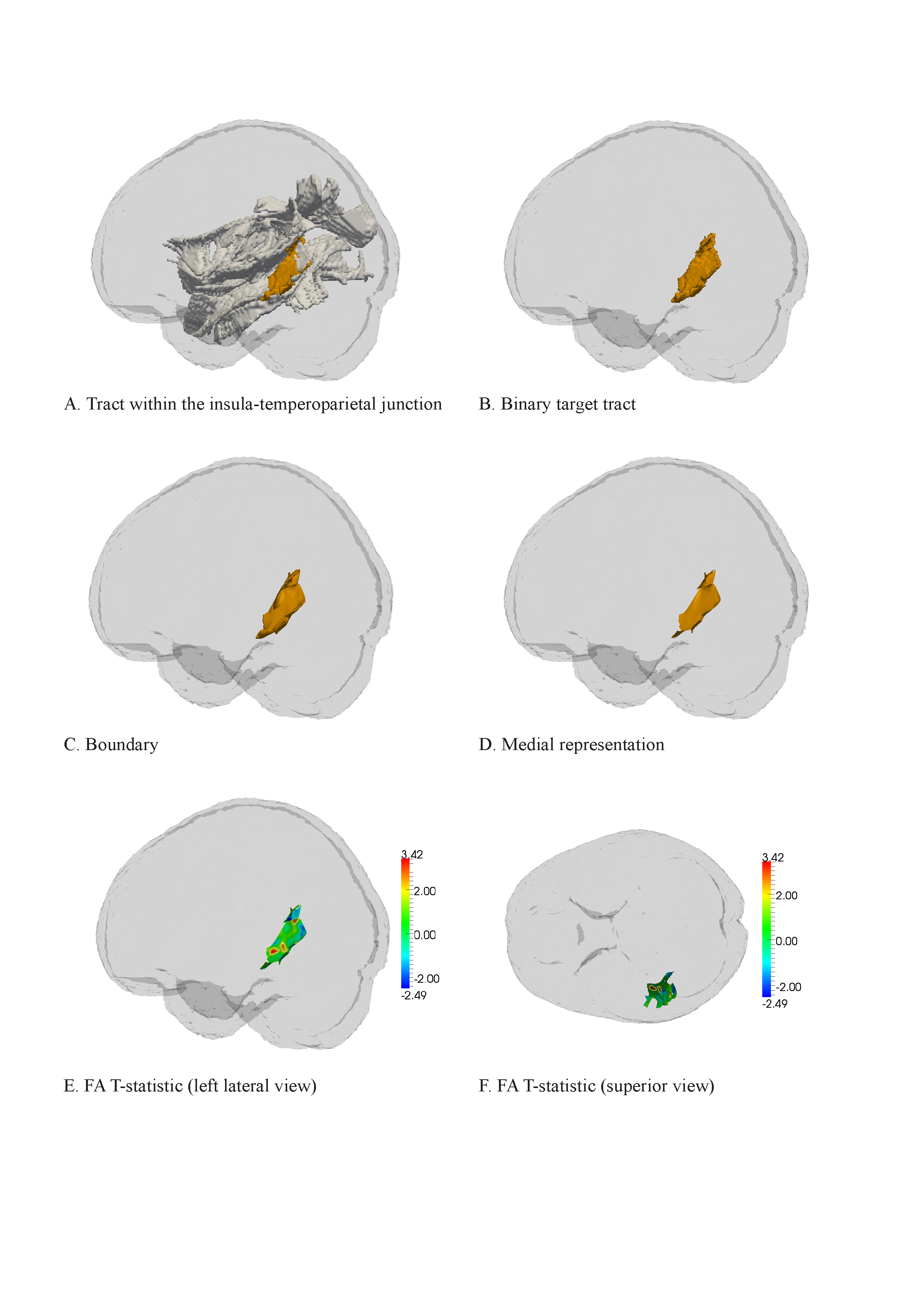

Supplement: Figure S4 — Short association fibres connecting the banks of the superior temporl sulcus to the middle temporal gyrus. (TIFF) [file pone.0112842.s004.tiff]

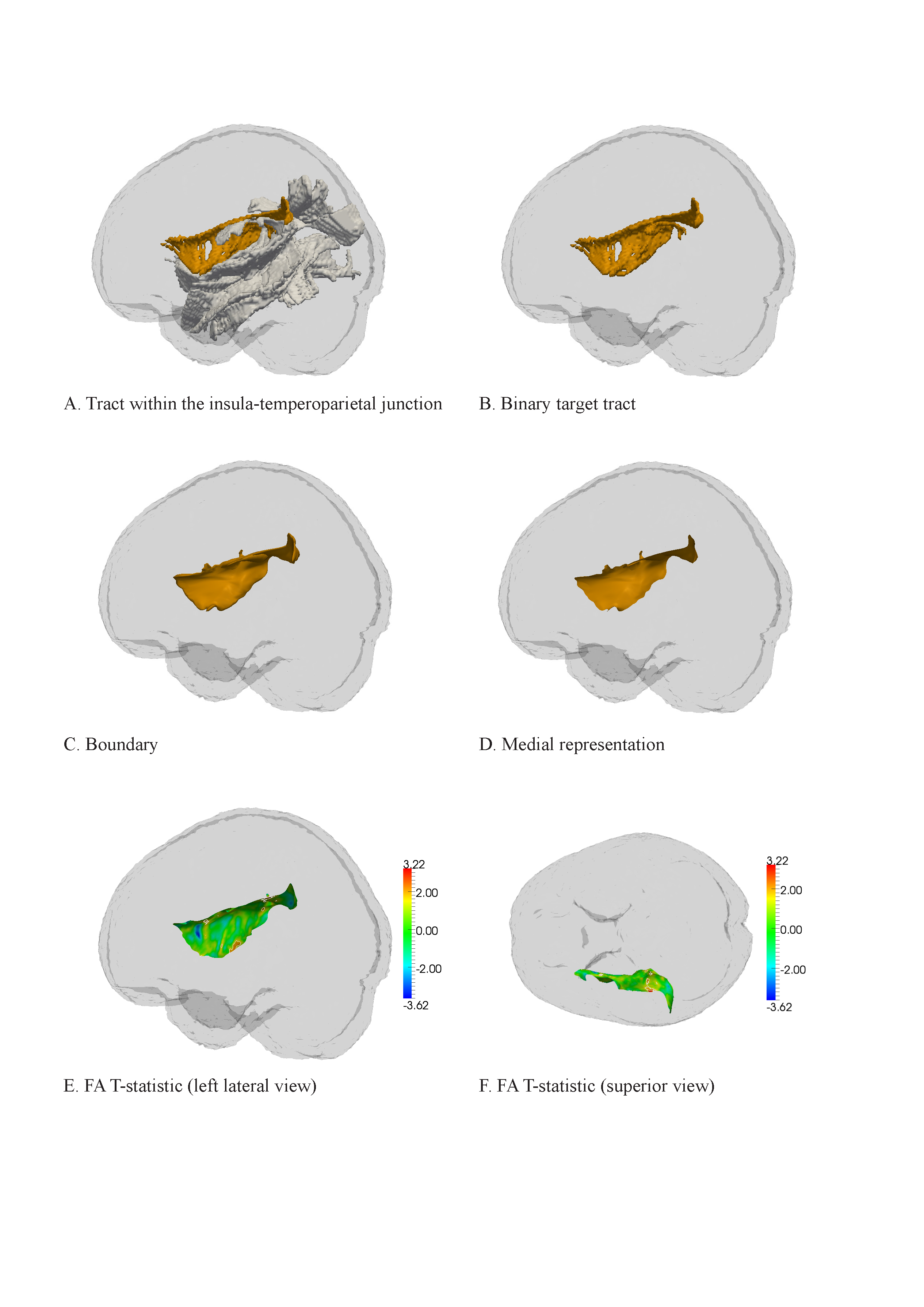

Supplement: Figure S5 — Short association fibres connecting the insula cortex to the supramarginal gyrus. (TIFF) [file pone.0112842.s005.tiff]

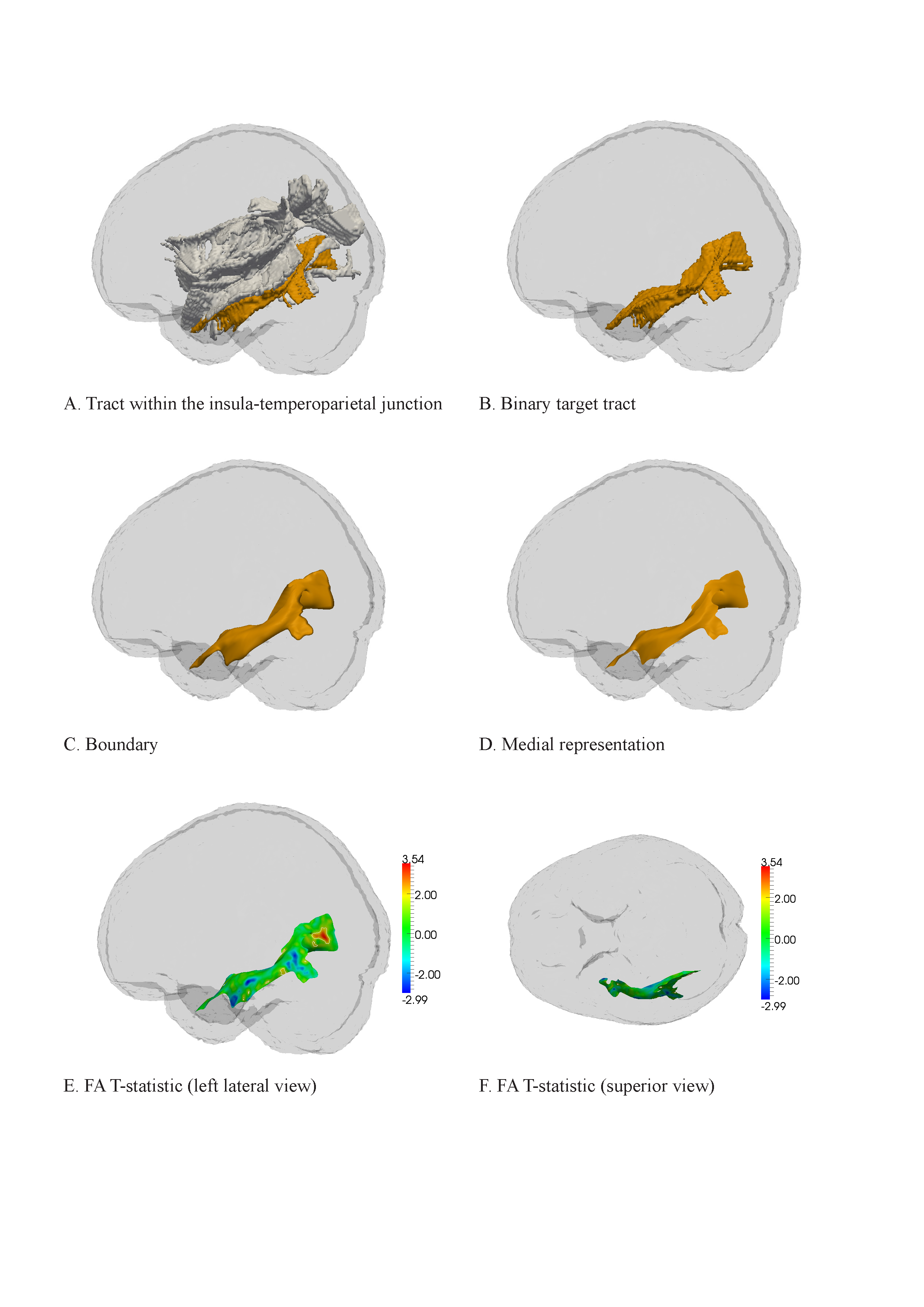

Supplement: Figure S6 — Short association fibres connecting the insula cortex to the supramarginal gyrus. (TIFF) [file pone.0112842.s006.tiff]

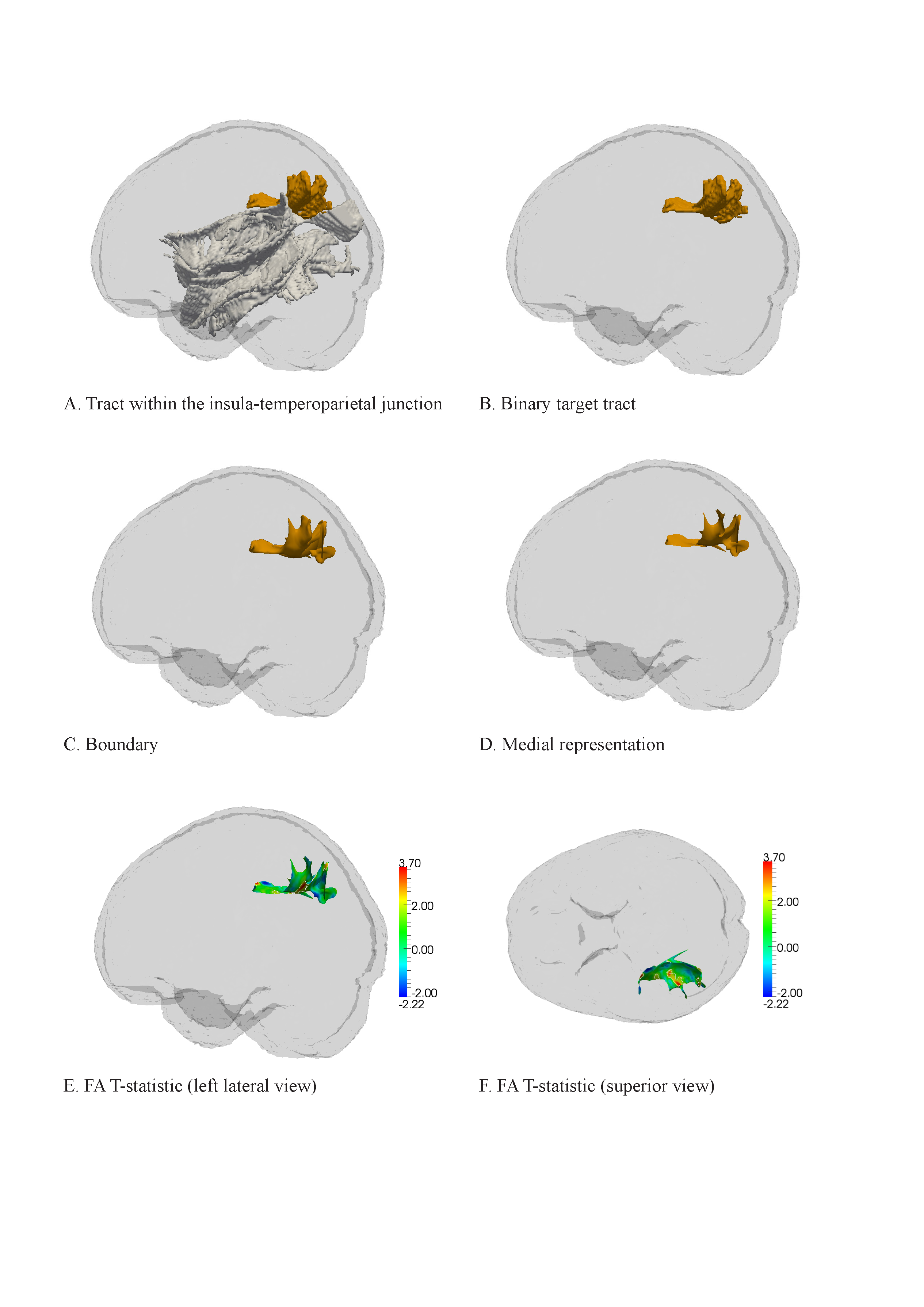

Supplement: Figure S7 — Short association fibres connecting the supramarginal gyrus to the inferior parietal region. (TIFF) [file pone.0112842.s007.tiff]

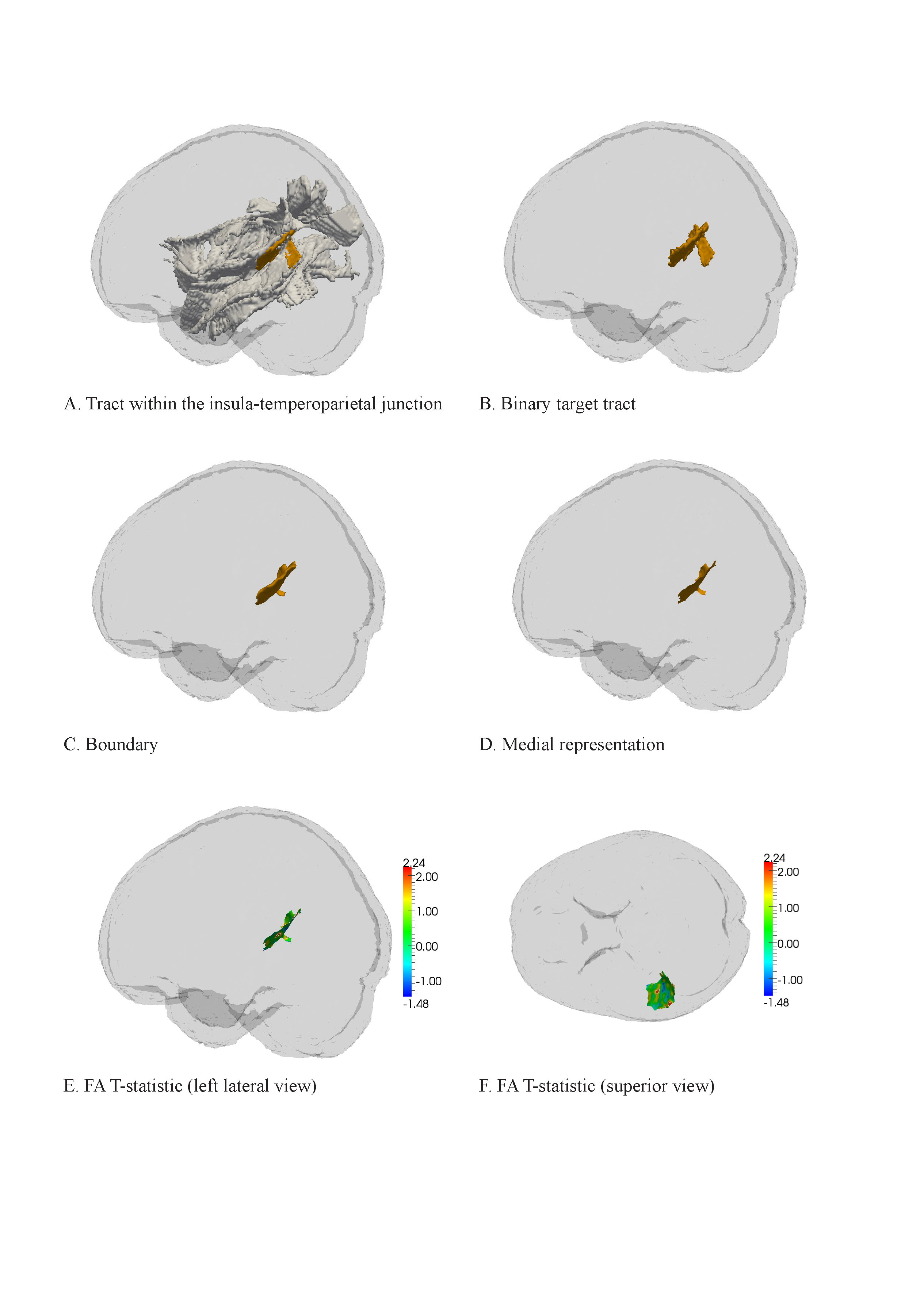

Supplement: Figure S8 — Short association fibres connecting the banks of the superior temporal sulcus to the superior temporal gyrus. (TIFF) [file pone.0112842.s008.tiff]

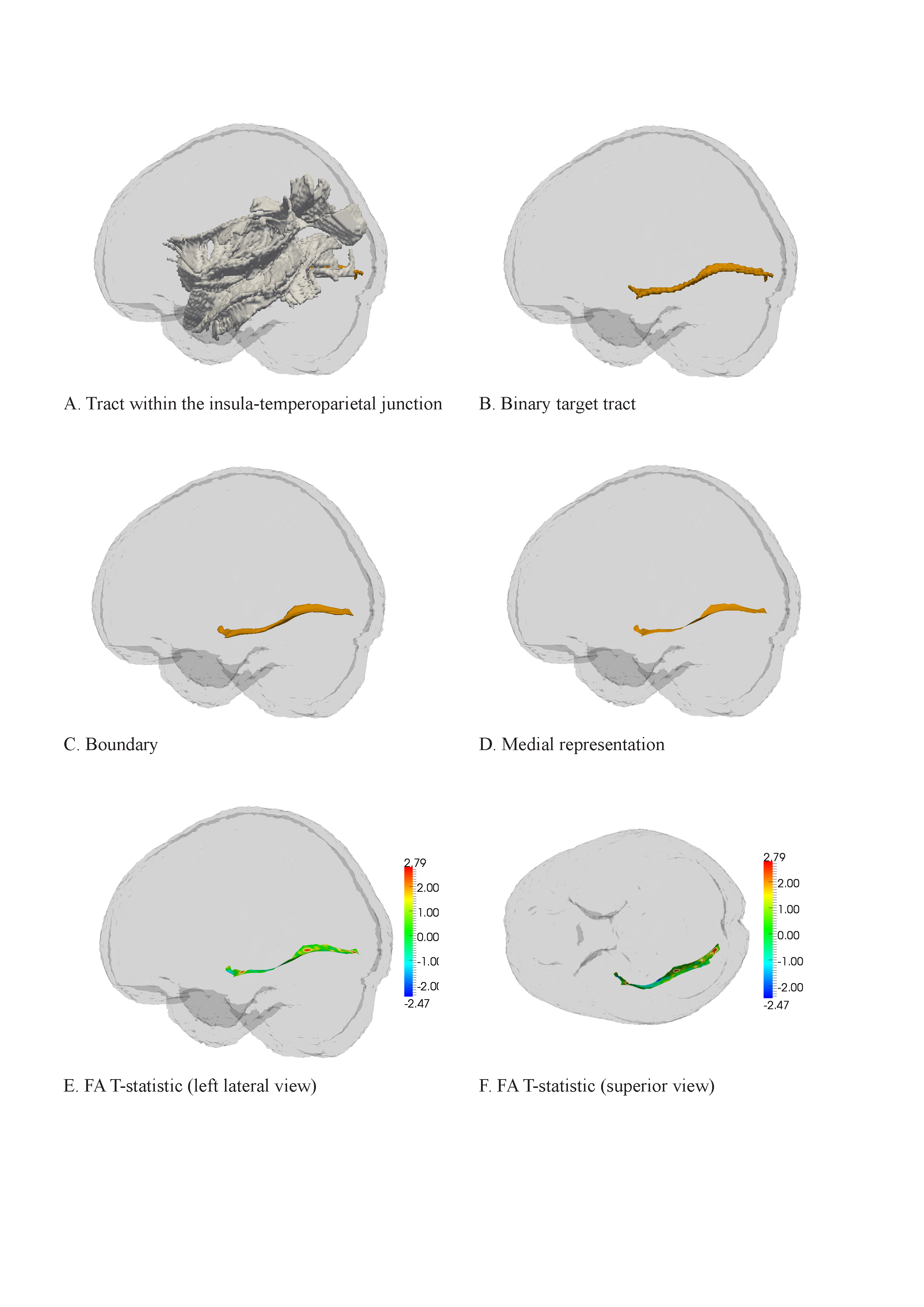

Supplement: Figure S9 — Short association fibres connecting the fusiform to the superior temporal gyrus. (TIFF) [file pone.0112842.s009.tiff]

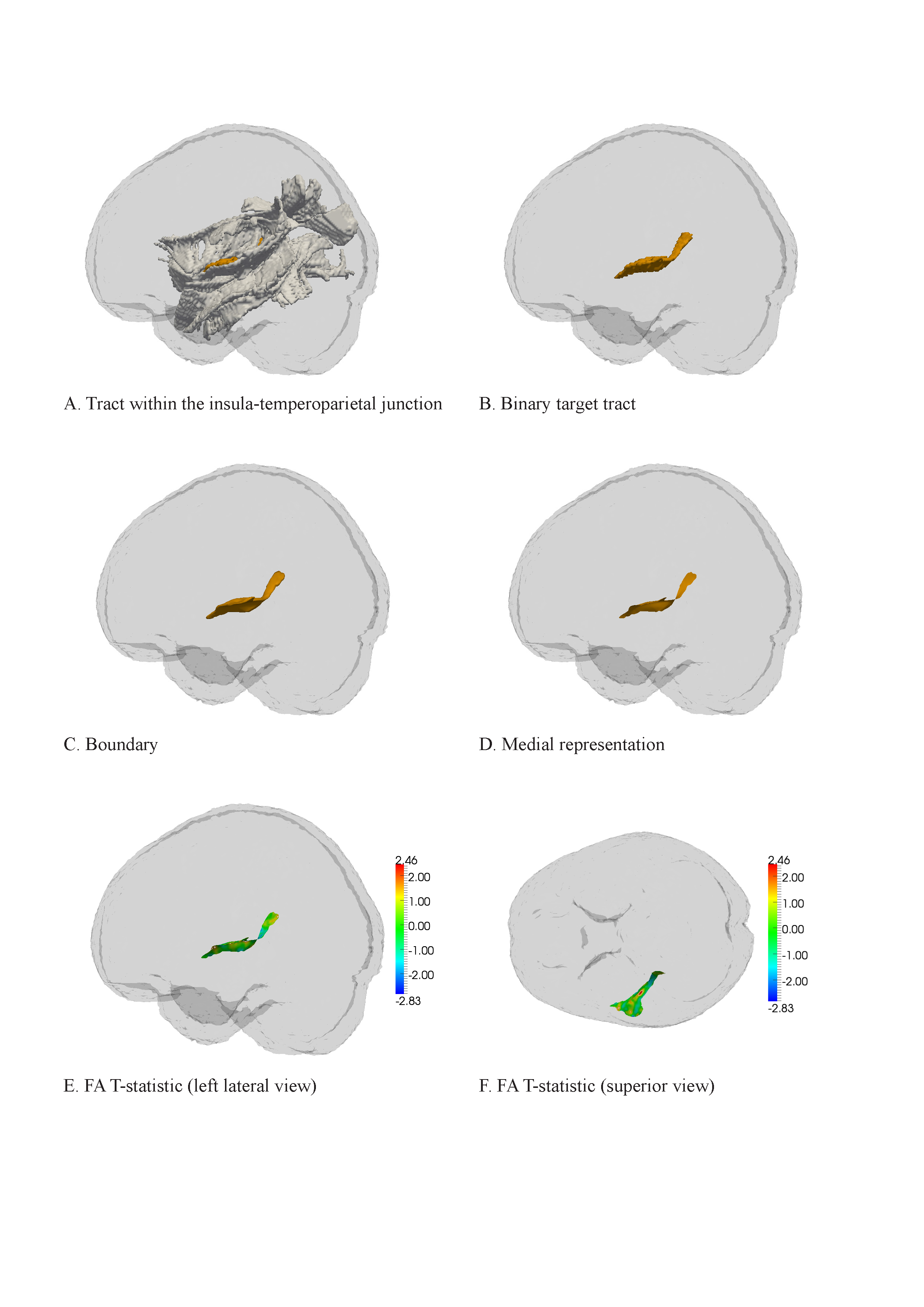

Supplement: Figure S10 — Short association fibres connecting the Heschls' gyrus to the superior temporal gyrus. (TIFF) [file pone.0112842.s010.tiff]

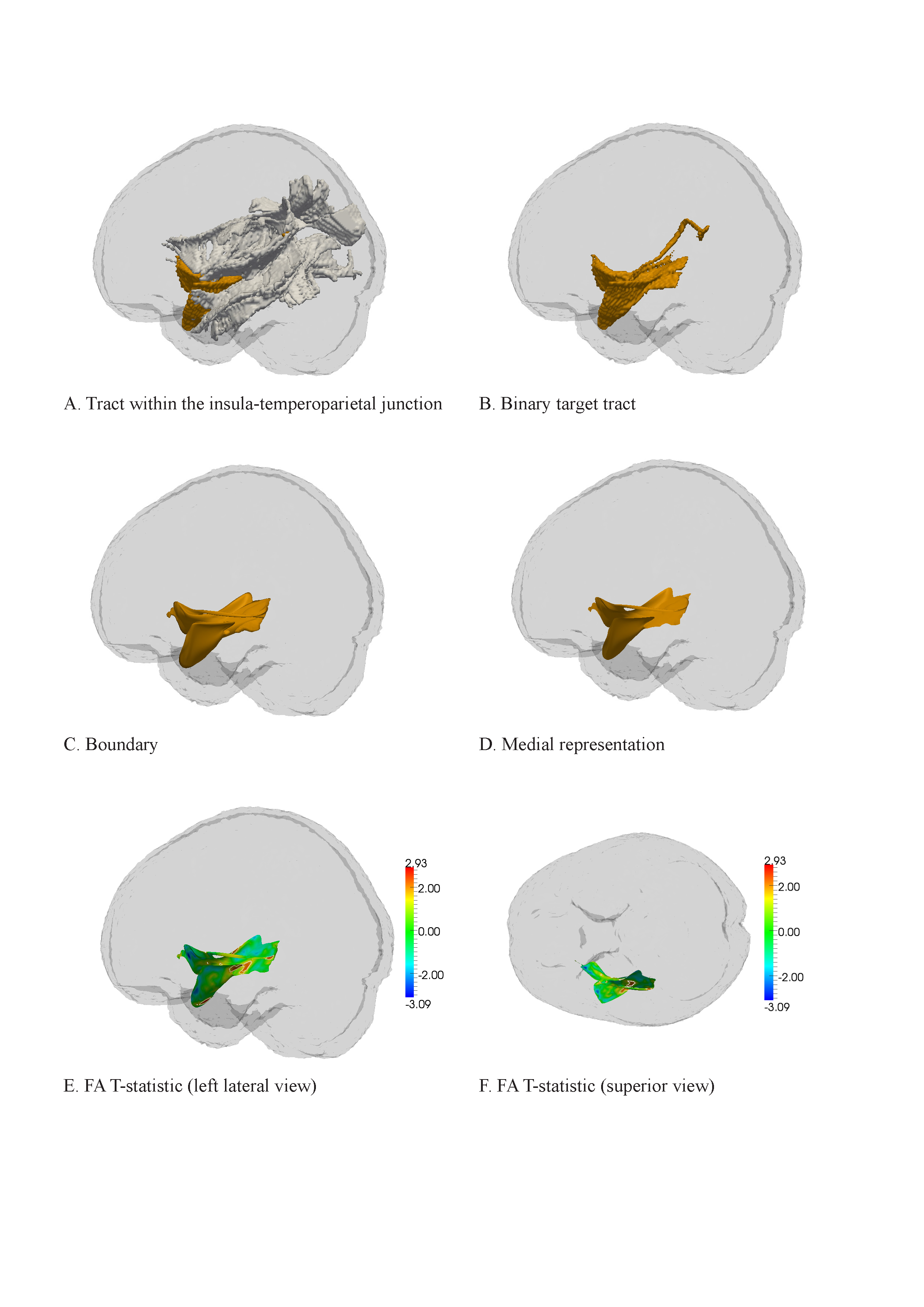

Supplement: Figure S11 — Short association fibres connecting the insula cortex to the superior temporal gyrus. (TIFF) [file pone.0112842.s011.tiff]

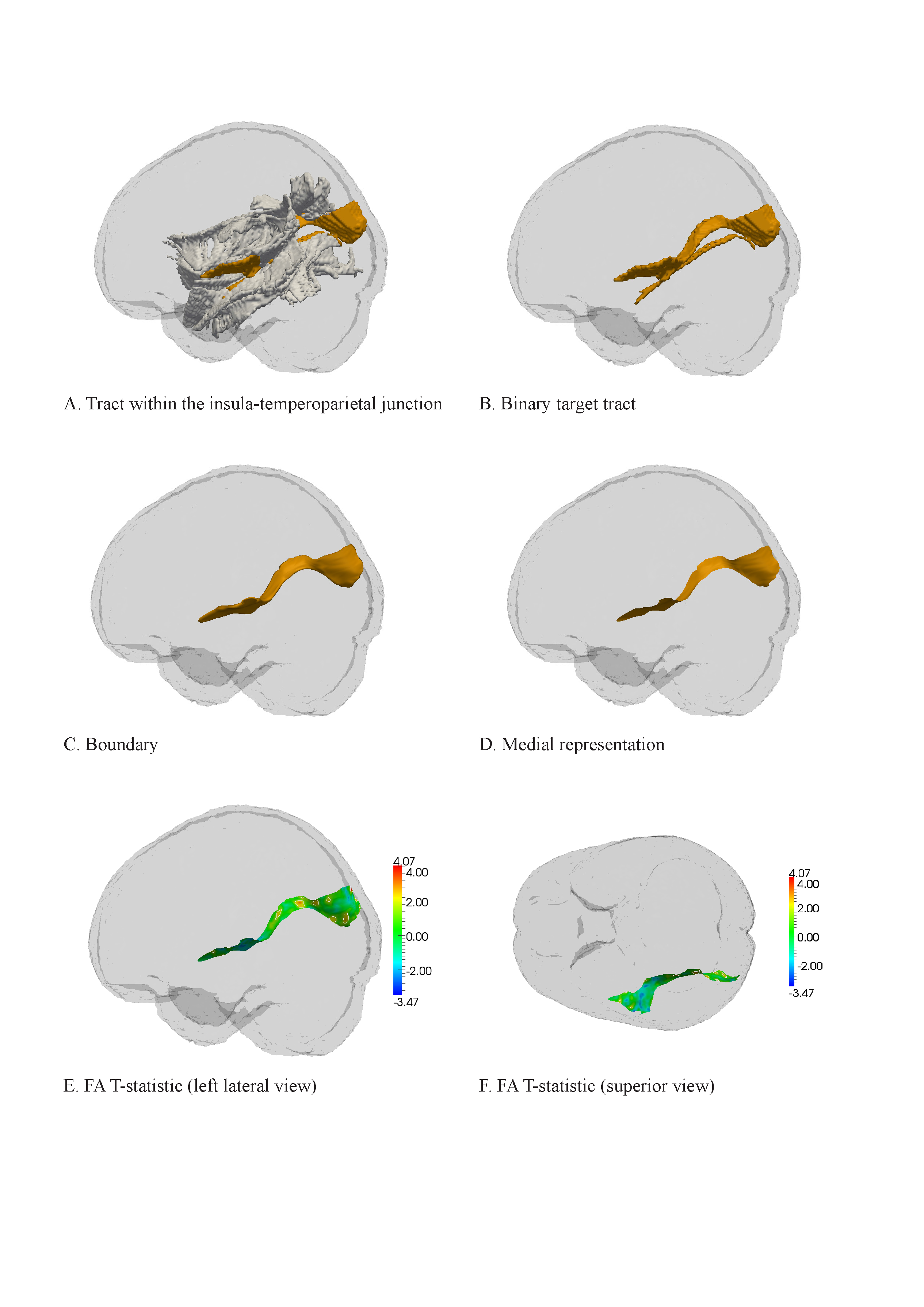

Supplement: Figure S12 — Short association fibres connecting the inferior parietal cortex to the superior temporal gyrus. (TIFF) [file pone.0112842.s012.tiff]

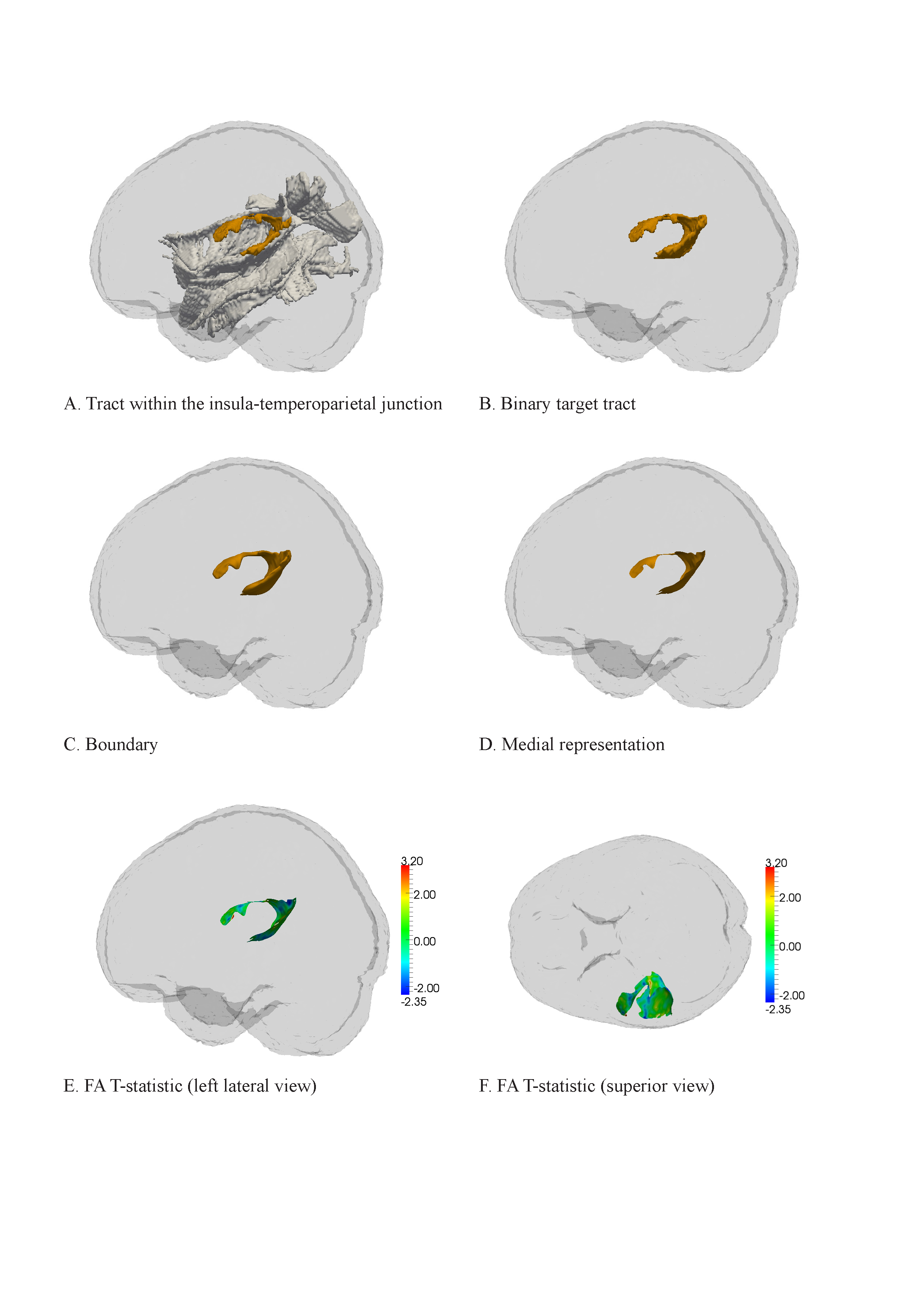

Supplement: Figure S13 — Short association fibres connecting the supramarginal gyrus to the superior temporal gyrus. (TIFF) [file pone.0112842.s013.tiff]
